# Dental Science in 1480, as Expounded by Peter De Largelata

**Published:** 1899-11

**Authors:** William H. Trueman

**Affiliations:** Philadelphia


### DENTAL SCIENCE IN 1480, AS EXPOUNDED BY PETER DE LARGELATA.

BY WILLIAM H. TRUEMAN, PHILADELPHIA.

A WORK by Peter de Largelata, Master Surgeon, of Bononia, is the oldest printed book containing an account of diseases of the teeth that I have yet met with. The copy before me is dated Venice, September 12, 1499, and is of the fourth edition. The Army Medical Library at Washington has a copy of an earlier edition noted in its catalogue, dated 1480.

The book contains two hundred and sixty-two folio pages of very compact type, equivalent to about five hundred octavo pages of a modern medical book; about five pages are given to diseases of the teeth. It is in Latin, and without illustrations. The title-page simply gives the name of the author and his title,—"Cirurgia Magistri Petri de Largelata;" the title-page as we now know it, giving at least an idea of the contents of the book, while not unknown, was not then in general use; it came a few years later. The catalogue of the Army Medical Library notes that the name is sometimes written "Argelata." I have been unable to gather any further information of the writer.

We may remember that the printed book was, on its advent, surreptitiously introduced as the work of the scribe, and was purposely made to conform as nearly as possible to those it supplanted. It was only when the market became glutted, and the price in consequence declined, that it became known as the product of the printing press. It was only when the art of printing had fully proved its superiority to the slow and inaccurate work of the scribe that the printed book began to assume, slowly, and step by step, the new dress its new mode of production rendered possible. The printed book, as a printed book, made its advent about 1430; it did not, however, assume any great commercial importance until about 1450, when Faust and Gutenberg, at Mayence, made printing an important business.

In this book, as was customary in manuscript copies, the matter later placed upon the title-page is found on the first page, with nothing to distinguish it in either type or arrangement from other portions of the text, and reads, translated, "The beginning of the First Book of the Complete Teacher of the Healing Art, by Peter de Largelata, Master Surgeon, of Bononia." The next paragraph may be considered a preface; it reads, "Introducing for the fourth time, so satisfactory they have proved to my fellow practitioners throughout the world, the four Canons of Avicenna, as ordinarily accepted." He then thanks his patrons for the favor shown by so often calling for his book, and proceeds to outline its contents. Bononia, now Bologna, is an ancient Italian city about eighty miles north of Florence, and was once an important educational centre.

It is by no means a record of original research. He follows closely his Arabian master. His description of disorders of the teeth are scarcely recognizable by a practitioner of the present day, while his remedies, with but few exceptions, seem to be selected more with reference to the patient's imagination than for any special virtue that they may have had. Its peculiar type, abbreviations, obsolete words, and occasional typographical errors, make it a difficult book to translate. It is probable that older works than this, treating of our science, may exist, as it is known that very soon after the printing-press was recognized it was used to duplicate works on science and medicine, and long before the introduction of movable type, collegiate text-books were printed by the xylographic or wood-block process.

